# Hypocholesterolemic effects of *Kluyveromyces marxianus* M3 isolated from Tibetan mushrooms on diet-induced hypercholesterolemia in rat

**DOI:** 10.1590/S1517-838246220131278

**Published:** 2015-06-01

**Authors:** Yuanhong Xie, Hongxing Zhang, Hui Liu, Lixia Xiong, Xiuzhi Gao, Hui Jia, Zhengxing Lian, Nengsheng Tong, Tao Han

**Affiliations:** 1 Beijing Laboratory of Food Quality and Safety, Faculty of Food Science and Engineering, Beijing University of Agriculture, Beijing, China, Beijing Laboratory of Food Quality and Safety, Faculty of Food Science and Engineering, Beijing University of Agriculture, Beijing, China.; 2 China Agriculture University, College of Animal Science & Technology, China Agricultural University, Beijing, China, College of Animal Science & Technology, China Agricultural University, Beijing, China.; 3 Beijing China Investment Corporation Clinical Laboratory, Beijing, China, Beijing China Investment Corporation Clinical Laboratory, Beijing, China.

**Keywords:** *Kluyveromyces marxianus*, hypercholesterolemia, high-cholesterol diet

## Abstract

To investigate the effects of *Kluyveromyces marxianus* M3 isolated from Tibetan mushrooms on diet-induced hypercholesterolemia in rats, female Wistar rats were fed a high-cholesterol diet (HCD) for 28 d to generate hyperlipidemic models. Hyperlipidemic rats were assigned to four groups, which were individually treated with three different dosages of *K. marxianus* M3+HCD or physiological saline+HCD via oral gavage for 28 d. The total cholesterol (TC), triglycerides (TG), high-density lipoprotein cholesterol (HDL-C), and low-density lipoprotein cholesterol (LDL-C) levels in the serum and liver of the rats were measured using commercially available enzyme kits. In addition, the liver morphology was also examined using hematoxylin and eosin staining and optical microscopy. According to our results, the serum and liver TC, TG, LDL-C levels and atherogenic index (AI) were significantly decreased in rats orally administered *K. marxianus* M3 (p <0.01), and the HDL-C levels and anti atherogenic index (AAI) were significantly increased (p <0.01) compared to the control group. Moreover, *K. marxianus* M3 treatment also reduced the build-up of lipid droplets in the liver and exhibited normal hepatocytes, suggesting a protective effect of *K. marxianus* M3 in hyperlipidemic rats.

## Introduction

Hypercholesterolemia is considered to be a risk factor of cardiovascular disease and is the leading cause of morbidity and mortality in many countries (Law *et al.*, 1994). Elevated serum cholesterol levels are widely recognized as a contributing risk factor for the development of cardiovascular diseases, such as atherosclerosis, coronary heart disease and stroke. It has been reported that a 1% reduction in serum cholesterol could reduce the risk of coronary heart disease by 2 to 3% (Manson *et al.*, 1992). The decrease in cholesterol levels could be achieved by appropriate food intake, such as low-cholesterol, low-fat diets (Lora *et al.*, 2007), dietary fiber (Jiménez *et al.*, 2008; Theuwissen *et al.*, 2008), and yogurts containing specific probiotics (Akalin *et al.*, 1997; Danielson *et al.*, 1989).

Recently, some studies have demonstrated that the hypocholesterolemic effects of probiotics have resulted in an increased interest in this treatment modality, which is less expensive and may be considered a "natural health remedy." Several studies evaluating this effect have found that some *lactobacilli* or *bifidobacteria* can exhibit hypocholesterolemic properties in animal models (Fukushima and Nakano 1996; Gilliland *et al.*, 1989; Nguyen *et al.*, 2007; Kumar *et al.*, 2011) and humans (Agerbaek *et al.*, 1995; Anderson and Gilliland 1999; Xiao *et al.*, 2003). However, the hypocholesterolemic mechanism of lactic acid bacteria is still no clearly understood, although the bacteria appear to contribute to increased fecal excretion of bile acids and thereby improved overall hepatic cholesterol homeostasis (Jeun *et al.*, 2010). Moreover, some reports have failed to show hypocholesterolemic effects of probiotics (Hatakka *et al.*, 2008; Simons *et al.*, 2006). Thus, additional studies are required to strengthen the proposed hypotheses and to improve our understanding of how bacteria affect cholesterol metabolism, which might result in the more appropriate use of probiotics.

Kefir has been widely recommended in western countries for consumption by healthy people to lower the risk of chronic diseases and has also been provided to some patients for the clinical treatment of a number of gastrointestinal and metabolic diseases, hypertension, and allergy (St-Onge *et al.*, 2002). Yogurt prepared from Tibetan mushrooms and milk has an extraordinary taste and provides excellent nutrition. Tibetan kefir has a granular structure due to the presence of symbiotic microorganisms, such as *Lactobacillus* and yeast (Simova *et al.*, 2002). In addition, kefir culture was reported to exhibit the ability to assimilate cholesterol in milk (Vujicic *et al.*, 1992). Furthermore, Liu *et al.* (2006) demonstrated the hypocholesterolemic effect of kefir milk in male hamsters fed with a cholesterol-enriched diet. However, St-Onge *et al.* (2002) obtained a conflicting result and reported that kefir consumption did not result in the lowering of plasma lipid concentrations, although kefir resulted in increasing fecal isobutyric, isovaleric, and propionic acids as well as the total amount of fecal short chain fatty acids.

Moreover, some researchers have found that kefir-fermented milk can decrease plasma cholesterol levels and can promote cancer resistance. Furthermore, it has antioxidant properties, including a role in immune regulation, and can help to protect against pathogenic bacteria and spoilage organisms, as well as assist in the conservation of predominant gastrointestinal probiotic flora (Abd El-Gawad *et al.*, 2005; Mathara *et al.*, 2008; Nguyen *et al.*, 2007; Akalin *et al.*, 1997). The objective of this study was to evaluate the effects of *Kluyveromyces marxianus* M3 yeast isolated from Tibetan mushrooms on lowering cholesterol in rats.

## Materials and Methods

### Microbial cultures

*K. marxianus* M3 was isolated from Tibetan mushrooms and was cultured by a resident of Baicheng, Jilin province, China (Liu *et al.*, 2005). M3 strains (1–2%) were inoculated into 10 mL potato lactose liquid medium and grown at 28 °C for 24. The culture was centrifuged and diluted with 0.9% saline water to obtain a preparation of 2.0 × 10^7^ cfu/mL.

### Animals, diets and experimental design

Forty female Wistar rats (aged 3 weeks) with a weight of 140 ± 10 g were obtained from the Academy of Military Medical Sciences (Beijing, China). All rats were individually housed at a constant temperature and humidity (18–24 °C, 60%) with a 12 h light/dark cycle. After 1 week of acclimatization, all of the rats were fed a high-cholesterol diet (78.8% basic diet, 1% cholesterol, 10% egg yolk, 10% lard, 0.2% cholate, w/w) for 28 d. In addition, the rats were randomly assigned to four groups (n = 10), respectively. Group (NM): normal rats fed a standard high-cholesterol diet and physiological saline (5 mL/kg); Group α (LD): normal rats fed a standard high-cholesterol diet and *K. marxianus* M3 (5 mL/kg); Group β (MD): normal rats fed a standard high-cholesterol diet and *K. marxianus* M3 (10 mL/kg); Group IV (HD): normal rats fed a standard high-cholesterol diet and *K. marxianus* M3 (20 mL/kg).

The rats were intragastrically administered for 28 d, and food and water consumption and body weight were recorded daily. At the end of the feeding period, all rats were anesthetized by isoflurane and sacrificed by cervical dislocation. The kidney, heart and liver were immediately excised, and the serum was separated from the blood. The liver, heart and kidney were excised, rinsed in ice-cold physiological saline, weighed, and then stored at −20 °C.

### Serum lipid analysis

The samples were allowed to stand for 10 min and then centrifuged at 3500 r/min for 15 min, where the sediment was subsequently discarded. The TC (total cholesterol), TG (triglyceride), HDL-C (high density lipoprotein-cholesterol), and LDL-C (low density lipoprotein-cholesterol) levels were analyzed using kits (Bio-technology and Science Incorporation) and a fully automatic biochemical analyzer (Hitachi, Japan). The atherosclerosis index (AI) was calculated as follows: AI = (total cholesterol - HDL cholesterol)/HDL cholesterol.

### Liver lipid analysis

Isolated livers were weighed after rinsing with phosphate-buffered saline and blotted dry with filter paper. Each liver was homogenized in 20 volumes of extraction solution (chloroform: methanol = 2:1; v/v) and agitated for 60 min at room temperature (Zhao *et al.*, 2012). Liver cholesterol and triacylglycerols were measured using the kits previously described.

### Morphology of liver

Fresh livers of rats were fixed with 4% paraformaldehyde for 24 h, gradually dehydrated in a graded series of ethanol, clarified in xylene, and embedded in paraffin wax. The hematoxylin and eosin stained livers were observed using an optical microscope (Wang *et al.*, 2013).

### Statistical analysis

All data were expressed as the mean ± SD. Statistical analysis was performed using SPSS 13.0 software. Differences between the groups were analyzed by One-Way ANOVA followed by Duncan's multiple range tests. Statistical significance was considered at p <0.01.

## Results

### Effect on plasma lipid profiles

The effects of *K. marxianus* M3 live yeast supplementation on the serum lipid levels of rats are presented in Table 1. The rats subjected to a high cholesterol diet or high cholesterol diet with *K. marxianus* M3 had no obvious difference in body weight (BW) during the entire 7 weeks of experiments. And high cholesterol diet dramatically increased the serum TC, TG and LDL-C levels of rats in NM group, which demonstrated the hyperlipidemic model was set up successfully. In addition, oral administration of *K. marxianus* M3 for 7 weeks significantly decreased (p <0.01) the serum TC, TG and LDL-C levels of rats compared with the NM groups. In contrast, the serum HDL-C levels in the *K. marxianus* M3 supplemented rats significantly increased (p <0.01, p <0.05) compared to that in NM group after 7 weeks of administration.

**Table 1:** Effect of *Kluyveromyces marxianus* M3 from Tibetan Kefir on plasma lipid profiles of rats ( ± s, n = 6).

	NM				LD				MD				HD			
				
	1 week	3 week	5 week	7 week	1 week	3 week	5 week	7 week	1 week	3 week	5 week	7 week	1 week	3 week	5 week	7 week
BW (g)	155.1 ± 5.1	204.9 ± 9.9	251.9 ± 16.9	273.2 ± 20.8	154.3 ± 7.3	204.4 ± 11.6	246.4 ± 14.6	268.1 ± 20.9	156.2 ± 6.8	206.5 ± 10.5	244.1 ± 15.9	268.3 ± 21.3	155.4 ± 8.6	205.5 ± 12.5	247.3 ± 17.3	272.2 ± 21.2
TC (mmol/L)	2.86 ± 0.96	3.18 ± 1.06	4.76 ± 1.50	4.94 ± 1.90	2.84 ± 0.75	3.07 ± 0.95	3.36 ± 1.26**	2.75 ± 0.76**	2.72 ± 0.70	3.34 ± 1.35	3.39 ± 1.41**	2.42 ± 0.62**	2.58 ± 0.60	3.81 ± 1.43	4.10 ± 1.82	3.17 ± 1.09**
TG (mmol/L)	0.39 ± 0.23	0.24 ± 0.20	0.37 ± 0.12	0.51 ± 0.23	0.30 ± 0.15	0.25 ± 0.18	0.33 ± 0.09	0.31 ± 0.07**	0.37 ± 0.21	0.41 ± 0.28	0.37 ± 0.11	0.36 ± 0.12**	0.33 ± 0.12	0.19 ± 0.13	0.34 ± 0.12	0.33 ± 0.12**
HDL-C (mmol/L)	0.70 ± 0.16	0.48 ± 0.06	0.45 ± 0.08	0.43 ± 0.14	0.70 ± 0.15	0.60 ± 0.20	0.52 ± 0.10	0.62 ± 0.18**	0.64 ± 0.14	0.51 ± 0.17	0.46 ± 0.10	0.50 ± 0.17^DD^	0.58 ± 0.14	0.52 ± 0.17	0.40 ± 0.10	0.45 ± 0.10^DD^
LDL-C (mmol/L)	0.64 ± 0.20	1.04 ± 0.37	1.57 ± 0.51	1.58 ± 0.61	0.65 ± 0.20	0.82 ± 0.25	0.69 ± 0.34**	0.63 ± 0.33**	0.56 ± 0.16	1.04 ± 0.43	0.74 ± 0.47**	0.88 ± 0.41**	0.64 ± 0.17	0.94 ± 0.54	0.95 ± 0.58**	1.08 ± 0.41**
AI	2.85 ± 0.35	5.67 ± 0.53	10.11 ± 0.96	10.11 ± 0.91	3.01 ± 0.24	4.01 ± 0.34	5.67 ± 0.52**	3.35 ± 0.25**	3.17 ± 0.26	5.67 ± 0.51	6.14 ± 0.46**	4.00 ± 0.25**	3.55 ± 0.21	6.14 ± 0.46	10.00 ± 0.90	6.14 ± 0.74**
AII	0.26 ± 0.03	0.15 ± 0.02	0.09 ± 0.01	0.09 ± 0.01	0.25 ± 0.04	0.20 ± 0.03	0.15 ± 0.02**	0.23 ± 0.04**	0.24 ± 0.04	0.15 ± 0.02	0.14 ± 0.02**	0.20 ± 0.04**	0.22 ± 0.05	0.14 ± 0.02	0.10 ± 0.01	0.14 ± 0.01**

Data represent mean ± SD (n = 6 for each group). ^1^BW: body weight (g), ^2^AI = (TC-HDL-C)/HDL-C, ^3^AAI=HDL-C/TC. TC: Total cholesterol; TG: Triglyceride; HDL-C: High-density lipoprotein cholesterol; LDL-C: Low-density lipoprotein cholesterol; AI: Atherogenic index; AAI: Anti atherogenic index; NM: High-cholesterol diet group; LD: High-cholesterol diet with Low dosage of *K. marxianus* M3 group; MD: High-cholesterol diet with middle dosage of *K. marxianus* M3 group; HD: High-cholesterol diet with high dosage of *K. marxianus* M3 group.

DD p <0.05 compared with the control group;

** p <0.01 compared with the control group.

Moreover, oral administration of various dosages of *K. marxianus* M3 showed different degrees of changes in serum lipid. The serum TC, TG, and LDL-C levels were the most reduced in the LD group, decreasing by 44.33%, 39.21% and 60.12%, respectively, whereas the serum HDL-C level improved by 44.18% compared to the NM group. In the MD group, the serum TC, TG, and LDL-C levels decreased by 51.01%, 29.41% and 44.3%, respectively, whereas the HDL-C level improved by 16.27%. Moreover, the serum levels of TC, TG and LDL-C in the HD group were decreased by 35.82%, 35.29% and 31.64%, respectively, and the HDL-C level increased by 4.65% (Table 1). As a result, oral administration of *K. marxianus* M3 in rats significantly decreased (p <0.01) the serum TC, TG, LDL-C levels and atherogenic index (AI), and significantly increased (p <0.01) the serum HDL-C levels and anti-atherogenic index (AAI) compared to the NM group.

### Effect on Liver lipid profiles

After 7 weeks of treatment, the liver lipid levels of rats were also examined. As shown in Table 2, oral administration of *K. marxianus* M3 for 7 weeks significantly decreased (p <0.01) liver TC, TG, LDL-C levels and the AI compared with the NM groups. In addition, the liver HDL-C levels and AII of the *K. marxianus* M3 treatment group were significantly higher (p <0.01) compared to the NM group. Moreover, oral administration of *K. marxianus* M3 at various dosages showed different degrees of decreases in liver TC, TG, LDL-C levels and increases in liver HDL-C levels. After 7 weeks of administration, the liver TG, TC, LDL-C levels in the MD group significantly decreased (p <0.01) by 36.00%, 22.92% and 52.94% compared with the NM group. Furthermore, the liver HDL-C contents in the LD group rats reached 0.31 ± 0.10 mmol/L, which was 72.22% higher than that in the NM group. As a result, *K. marxianus* M3 treatment in the LD group significantly decreased (p <0.01) the liver AI and increased (p <0.01) the liver AII compared with the NM group.

**Table 2:** Effect of Kluyveromyces marxianus from Tibetan Kefir on hepatic lipid profiles of rats ( ± s, n = 10).

	TC (mmol/L)	TG (mmol/L)	HDL-C (mmol/L)	LDL-C (mmol/L)	AI	AAI
NM	0.25 ± 0.07	0.48 ± 0.09	0.18 ± 0.07	0.17 ± 0.13	0.37 ± 0.07	0.73 ± 0.11
LD	0.16 ± 0.05**	0.37 ± 0.07**	0.31 ± 0.10**	0.08 ± 0.05**	−0.50 ± 0.07**	2.11 ± 0.11**
MD	0.19 ± 0.04**	0.42 ± 0.11^DD^	0.27 ± 0.11**	0.13 ± 0.04^DD^	−0.32 ± 0.04**	1.51 ± 0.09**
HD	0.20 ± 0.05**	0.41 ± 0.08^DD^	0.25 ± 0.13**	0.14 ± 0.05^DD^	−0.23 ± 0.06**	1.33 ± 0.10**

Data represent mean ± SD (n = 10 for each group). AI = (TC-HDL-C)/HDL-C, AAI=HDL-C/TC. TC: Total cholesterol; TG: Triglyceride; HDL-C: High-density lipoprotein cholesterol; LDL-C: Low-density lipoprotein cholesterol; AI: Atherogenic index; AAI: Anti atherogenic index; NM: High-cholesterol diet group; LD: High-cholesterol diet with Low dosage of *K. marxianus* M3 group; MD: High-cholesterol diet with middle dosage of *K. marxianus* M3 group; HD: High-cholesterol diet with high dosage of *K. marxianus* M3 group.

DD p <0.05 compared with the control group;

** p <0.01 compared with the control group.

### Effects on viscera organs

As shown in Table 3, a high cholesterol diet increased the heart, liver and kidney weight of the rats. After 7 weeks of administration, the viscera weight (heart, liver and kidney weight) and viscera coefficients in the rats of the LD group were significantly lower (p <0.01) compare to the NM group. The heart weight, heart coefficient, liver weight and liver coefficient in the MD and HD groups were also significantly decreased (p <0.01) compared to the NM group. Moreover, the kidney weight and kidney coefficient in the MD and HD groups were significantly decreased (p <0.05) compared to the NM group.

**Table 3:** Effect of *Kluyveromyces marxianus* from Tibetan Kefir on visceral weight and visceral coefficient of rats ( ± s, n = 10).

	Heart weight (g)	Cardiac coefficient (%)	Liver weight (g)	Liver coefficient (%)	Renal weight (g)	Renal coefficient (%)
NM	1.28 ± 0.17	0.48 ± 0.04	12.56 ± 1.72	4.81 ± 0.21	1.73 ± 0.17	0.64 ± 0.06
LD	0.81 ± 0.11**	0.28 ± 0.02**	8.86 ± 1.14**	3.24 ± 0.14**	1.47 ± 0.11**	0.54 ± 0.04**
MD	0.85 ± 0.15**	0.30 ± 0.03**	9.23 ± 1.37**	3.43 ± 0.17**	1.54 ± 0.14^DD^	0.57 ± 0.02^DD^
HD	0.87 ± 0.13**	0.33 ± 0.04**	9.31 ± 1.21**	3.45 ± 0.16**	1.60 ± 0.18^DD^	0.60 ± 0.03^DD^

Data represent mean ± SD (n = 10 for each group). Cardiac coefficient = heart weight/body weight; Liver coefficient = liver weight/body weight; Renal coefficient = renal weight/body weight; NM: High-cholesterol diet group; LD: High-cholesterol diet with Low dosage of *K. marxianus* M3 group; MD: High-cholesterol diet with middle dosage of *K. marxianus* M3 group; HD: High-cholesterol diet with high dosage of *K. marxianus* M3 group.

DD p <0.05 compared with the control group;

** p <0.01 compared with the control group.

**Figure 1:**
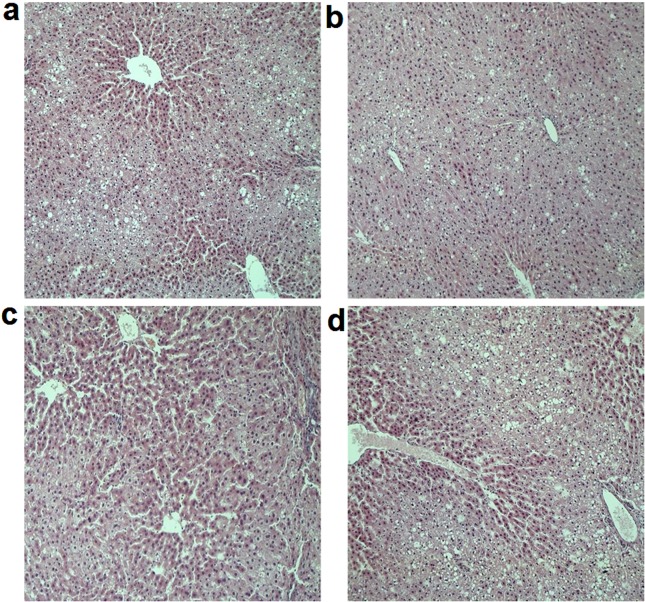
Histology of liver steatosis in rats. A: high-cholesterol diet group; B: high-cholesterol diet+*K. marxianus* M3 (5 mL/kg); C: high-cholesterol diet+*K. marxianus* M3 (10 mL/kg); D: high-cholesterol diet+*K. marxianus* M3 (20 mL/kg). All the photomicrographs show HE staining (original magnification × 100).

We further examined the hepatic morphology in rats. As shown in Figure 1a, in the NM group rats, the structure of the hepatic lobule had disappeared, and the liver cell morphology was irregular. There were different degrees of edema, focal necrosis, and fatty degeneration of liver cells. Moreover, the liver cells exhibited massive fatty changes and severe steatosis with cytoplasmic vacuoles, and the infiltration of inflammatory cells were visible. Taken together, these conditions suggested damage due to a high-cholesterol diet on the hepatic cells. In contrast, the size of the lipid droplets in the LD group was remarkably smaller than those in the NM group (Figure 1b), and the hepatic cells exhibited normal histology. In addition, the lipid droplets in the MD and HD groups were also reduced in varying degrees (Figure 1c, d). Taken together, our results indicated that *K. marxianus* M3 treatment reduced the build-up of lipid droplets and maintained normal hepatocytes.

## Discussion

Recently, considerable attention has focused on the potential of probiotics in altering lipid metabolism. This interest stems from growing evidence that probiotics reduce the concentration of cholesterol in vivo (Mohan *et al.*, 1995; Abdulrahim *et al.*, 1996; Panda *et al.*, 2003; Nguyen *et al.*, 2007; Wang *et al.*, 2009; Alkhalf *et al.*, 2010; Wang *et al.*, 2013). Generally, a high-cholesterol diet can increase body weight (Xie *et al.*, 2011). According to our results, addition of *K. marxianus* M3 live yeast with high-cholesterol diet did not significantly change the body weight of rats. However, *K. marxianus* M3 treatment for 7 weeks significantly decreased (p <0.01) the serum TC, TG and LDL-C levels in rats. In particular, these effects were more evident in the LD group (TC, TG and LDL-C reduced by 51.01%, 39.22% and 60.13%, respectively) (Table 1). Our results indicated that there was a relationship between the formation and reduction of the metabolism of cholesterol in the serum. Similar results were reported for the cholesterol-reducing activity of yeast (Yalçin *et al.*, 2008; Yalçin *et al.*, 2009), *Lactobacillus* (Nielson and Gilliland, 1985, Gilliland *et al.*, 1985; Hashimoto *et al.*, 1999; Simons *et al.*, 2006; Nguyen *et al.*, 2007; Xie *et al.*, 2011; Wang *et al.*, 2013) and *Bacillus* (Fukushima and Nakao, 1995).

High concentrations of TC and LDL-C are strongly associated with an increased risk of coronary heart disease. A reduction in TC and LDL-C in a hypercholesterolemic individual can reduce the incidence of cardiovascular disease (Probstfield and Rifkind 1991). Moreover, elevated levels of oxidized LDL-C are associated with artherosclerotic plaque formation on the artery walls, but increased HDL-C levels may reduce the risk due to the ability of HDL to transport cholesterol back to the liver for excretion or to other tissues of cardiovascular disease (Lewis *et al.*, 2005). According to our results, *K. marxianus* M3 supplementation dramatically increased the serum HDL-C level (p <0.01, p <0.05) in rats (Table 1). As a result, the AI of the *K. marxianus* M3 treatment groups was significantly decreased (p <0.01) compared to the NM group. Thus, we confirmed that *K. marxianus* M3 exerted a hypolipidemic effect and could alleviate lipid related metabolic syndrome. Similar results were reported by Hashimoto *et al.* (1999), in which a diet containing *L. casei* TMC 0409 increased the concentration of HDL-C in blood, which was consistent with other studies (Akalin *et al.*, 1997; Danielson *et al.*, 1989; De Smet *et al.*, 1998). However, conflicting results were reported (Chiu *et al.*, 2006; St-Onge *et al.*, 2002; Keim *et al.*, 1981, Rossouw *et al.*, 1981; De Roos *et al.*, 1998) in humans and animals.

In general, cholesterol is indispensable to the human body, and its levels are subjected to complex regulation. Cholesterol is modified into oxysterols, including 22- and 24-hydroxy cholesterol, when excess cholesterol is deposited in hepatic cells (Satoshi Hirako *et al.*, 2011). As expected, we demonstrated that the high-cholesterol diet increased hepatic TC, TG and LDL-C levels in rats (Table 2). Rats supplemented with *K. marxianus* M3 displayed significant reductions in hepatic TC, TG and LDL-C levels. These findings demonstrated that the serum cholesterol and TG levels in *K. marxianus* M3-treated rats were reduced, rather than merely being redistributed from the blood to the liver. Moreover, our results were consistent with previous reports (Kumar *et al.*, 2011; Chiu *et al.*, 2006).

In this study, a high-cholesterol diet promoted the visceral weight (heart, liver and renal) in rats (Table 3). In addition, oral administration of *K. marxianus* M3 significantly reduced the visceral weights and visceral coefficients, suggesting a protection of *K. marxianus* M3 to the visceral organs under a high-cholesterol diet. Moreover, the histology of liver steatosis also supported this result. High-cholesterol diet caused different degrees of edema, focal necrosis, and fatty degeneration of liver cells (Figure 1a). In contrast, the *K. marxianus* M3 treatment could reduce the build-up of lipid droplets and maintained normal hepatocytes (Figure 1b, c). The result of liver tectology proved that the *K. marxianus* M3 had important potential in alleviating hepatic steatosis attributed to mediation of lipid metabolism and had protective effects on hepatic structure. Similar results have also been reported (Wang *et al.*, 2013; Xie *et al.*, 2011).

In recent yeas, several hypotheses have been proposed to explain the hypocholesterolemic effects of the probiotic strains: (1) consumption or absorption of cholesterol by probiotic strains (Pigeon *et al.*, 2002; Liong and Shah 2005); (2) the cholesterol is converted into coprostanol by cholesterol reductase, which is produced by probiotic strains (Lye *et al.*, 2010); (3) some probiotic strains excrete bile salt hydrolase, leading to increased bile excretion in feces (Begley *et al.*, 2010), etc. There are some reports on bile salt hydrolase in different species of *Lactobacillus*, *Enterococcus*, *Peptostreptococcus*, *Bifidobacterium*, *Clostridium*, and *Bacteroides* (Liong and Shah 2005; Begley *et al.*, 2010). In our previous research, we have cloned the bile salt hydrolase (*bsh*) gene in *K. marxianus* (Genebank Acession: JQ247427.1). So we proposed that the hypocholesterolemic effects of *K. marxianus* M3 might cased by the activity of bile salt hydrolase. And further research could be conduct in this field.

In conclusion, our results suggested that *K. marxianus* M3 is a safe probiotic with the potential to reduce serum cholesterol and triglyceride levels. Thus, further studies are required to determine the mechanism underlying the cholesterol-lowering effect. It will also be necessary to test more animals, utilizing varying doses of *K. marxianus* M3 over longer time periods, to assess the long-term probiotic potential of *K. marxianus* M3.
